# Nanopore direct RNA sequencing reveals transmissible gastroenteritis virus epitranscriptomic and transcriptomic landscapes modulated by gene 7

**DOI:** 10.1099/mgen.0.001684

**Published:** 2026-04-29

**Authors:** Qingqiu Jiang, Zhihao Guo, Lu Tan, Yanwen Shao, Guoqian Gu, Xiaomin Zhao, Haidong Wang, Runsheng Li

**Affiliations:** 1 Department of Infectious Diseases and Public Health, Jockey Club College of Veterinary Medicine and Life Sciences, City University of Hong Kong, Kowloon, Hong Kong, SAR; 2 College of Veterinary Medicine, Northwest A&F University, No. 22 Xinong Road, Yangling, Shaanxi Province, 712100, PR China; 3 School of Public Health and Emergency Management, Southern University of Science and Technology, Shenzhen, PR China; 4 Tung Biomedical Sciences Centre, City University of Hong Kong, Hong Kong, PR China; 5 Department of Precision Diagnostic and Therapeutic Technology, City University of Hong Kong, Matter Science Research Institute (Futian), Shenzhen, Guangdong, PR China

**Keywords:** epitranscriptome, innate immunity, m6A regulatory factor, N6-methyladenosine (m6A), polyA, TGEV gene 7

## Abstract

Viral non-structural proteins are key mediators of host–virus interplay, including RNA modification dynamics. The function of the transmissible gastroenteritis virus (TGEV) gene 7, which encodes a non-structural protein, remains poorly understood. Using Oxford Nanopore direct RNA sequencing, we explored the host and viral RNA landscapes modulated by TGEV gene 7 in Swine testis cells. Deletion of the TGEV gene 7 halved viral RNA replication yet significantly increased m6A modification levels on both the viral genome and host mRNAs. This epitranscriptomic rewiring was accompanied by reciprocal shifts in the m6A regulators FTO (eraser) and RBM15 (writer). Despite comparable bulk transcriptome changes, gene 7 deletion introduced additional differentially expressed genes, showing stronger enrichment of antiviral and chemokine pathways, indicating heightened innate immunity. PolyA analysis uncovered a gene 7-dependent extension of viral, but not host, polyA tails. These findings highlight RNA-modification machinery as a potential target for coronavirus control and provide a framework for vaccine strategies exploiting gene 7 attenuation.

**Impact Statement**

Transmissible gastroenteritis virus causes acute, highly contagious intestinal disease in pigs, characterized by vomiting, diarrhoea and dehydration, with mortality rates approaching 100% in neonatal piglets, leading to substantial economic losses in the swine industry. Beyond its clinical impact, TGEV holds critical importance as a model coronavirus in scientific research. Its study provides fundamental insights into coronavirus biology, directly informing the development of broad-spectrum antiviral drugs and serving as an early-warning system for potential zoonotic coronavirus emergence. Our investigation into the function of the TGEV gene 7, therefore, holds significant implications for understanding coronavirus pathogenesis, developing novel therapeutics and enhancing pandemic preparedness.

## Data Summary

The raw nanopore sequencing data (FAST5 files) generated in this study have been deposited in the BioProject under the public accession code PRJNA1256614. All other supporting data are provided in the supplementary materials.

## Introduction

Transmissible gastroenteritis virus is a surrogate of the coronavirus, which causes acute, highly contagious intestinal disease in pigs, characterized by vomiting, diarrhoea and dehydration, with mortality rates approaching 100% in neonatal piglets, leading to substantial economic losses in the swine industry [1,4]. The causative agent is transmissible gastroenteritis virus (TGEV), an enveloped *Alphacoronavirus*. TGEV possesses a large, positive-sense single-stranded RNA genome of ~28.5 kb, comprising nine ORFs arranged in the order of 5′ UTR-ORF1a-ORF1b-S-ORF3a-ORF3b-E-M-N-ORF7-3′ UTR [5,6].

Following the entry of TGEV, the positive-sense genomic RNA (gRNA) is synthesized as a full-length negative template and serves as a template for the synthesis of progeny viral RNA [7]. Additionally, a nested set ofsubgenomic RNAs (sgRNAs) is transcribed, each containing a common 5′ leader sequence and the body sequence encoding one ORF and the rest of the gRNA till 3′ UTR [7]. The gRNA and sgRNAs are used to translate structural proteins, including spike (S), envelope (E), membrane (M) and nucleocapsid (N) proteins, which are essential for virion assembly and the viral infection process [8,9]. In addition, TGEV encodes several non-structural and accessory proteins, including ORF1a, ORF1b, ORF3a, ORF3b and ORF7 [10]. Notably, coronavirus non-structural proteins have been reported to influence viral virulence, host immune modulation, replication efficiency and RNA modifications [11,12].

N6-Methyladenosine (m6A) is the most abundant internal mRNA modification in eukaryotic cells, known to regulate mRNA stability, splicing and translation efficiency [13,14]. The abundance of host m6A is dynamically regulated by the writers (METTLs and RBM15), erasers (FTO) and readers [13]. Increasing evidence suggests that during infection, viruses induce alterations in host m6A, which in turn promote viral replication [15,16]. And disruptions in host m6A writers pose a great impact on the survival of the virus [17,18]. The m6A modification has become a key feature in studying virus–host interaction [19]. However, the detection of m6A sites in both host and viral RNAs is still hard for methods developed based on short-read sequencing [20]. Nanopore direct RNA sequencing records the current changes when the full-length RNA molecule passes through the nanopore, and the electrical signal can be converted into the nucleotide sequence. In many cases, modified ribonucleotides will cause changes in ion signals and affect the accuracy of downstream base calls [21]. Several methods were developed exploiting changes in current or increased error rates to identify modified RNA sites [22]. The host DRACH (D=A/G/U, R=A/G, A=the m6A-modified base, H=A/C/U) motif, the canonical consensus sequence for m6A deposition, can be detected by m6Anet with robust performance in identifying single-molecule m6A modifications (area under the receiver operating characteristic curve [ROC AUC] = 0.90; area under the precision–recall curve [PR AUC] = 0.91) [23]. And the viral RNA modification can also be profiled with a fine-tuned cutoff from a negative control [24].

The TGEV ORF 7 only contains one gene (gene 7), which encodes a non-structural protein consisting of 78 amino acids, with a molecular weight of ~9.1 kDa [10,25]. Although not essential for virus viability, gene 7 has been reported to help the virus antagonize host antiviral responses [25,26]. Also, gene 7 is directly linked to the 3′ UTR of all TGEV gRNA and sgRNAs, suggesting a potential role in regulating their expression [27]. To explore the potential regulatory role of TGEV gene 7, the development of RNA modification detection technology has been crucial, as it now enables the profiling of modifications in both the host and viral genomes [15,28]. Given the emerging role of RNA modifications in viral infections and the undefined molecular function of TGEV gene 7, we hypothesized that gene 7 acts as a viral modulator of the host epitranscriptomic landscape, specifically to suppress m6A-mediated antiviral defenses. To test this, we asked whether ablation of gene 7 would (1) alter the expression of key m6A regulators, (2) increase m6A deposition on host and viral RNAs, (3) attenuate viral replication and (4) potentiate the innate immune response. We employed Nanopore direct RNA sequencing as a powerful tool to simultaneously profile the transcriptomic and epitranscriptomic landscapes, thereby providing a systems-level validation of our hypothesis.

Our results provided a comprehensive overview of how the TGEV gene 7 modulated m6A modification of both host and virus. Additionally, we found that the TGEV gene 7 significantly influences virus replication and the length of viral polyA tails. Furthermore, we identified related cellular pathways that were regulated by TGEV gene 7 through its impact on RNA modification systems. This result offers novel insights into the role of TGEV gene 7 in virus–host interactions. Generally, our findings underscore the importance of gene 7 in TGEV pathogenesis and suggest potential targets for antiviral strategies or vaccine design [29].

## Methods

### Cells and viruses

Swine testis (ST) cells were purchased from the American Type Culture Collection. ST cells were grown at 37 °C with 5% CO_2_ in Minimum Essential Medium alpha supplemented with 10% fetal bovine serum, vitamins, l-glutamine and penicillin–streptomycin.

We obtained wild-type TGEV (hereinafter referred to as TGEV-wt) and a recombinant TGEV, which had a deleted gene 7 (hereinafter referred to as TGEV-∆7) from Northwest Agriculture and Forestry University.

### Virus infection and RNA isolation

ST cells were seeded into 6-well plates at a density of 5×10^5^ cells per well and infected with viruses at an MOI of 1. After 1 h of incubation, the cells were washed with PBS three times and covered with a 1-ml cell culture medium, followed by further incubation. At 16 h post-infection, the supernatant was removed, and 800 µl TRIzol Reagent (Invitrogen, Waltham, MA, USA) was added to the cell monolayer. Phase separation and total RNA precipitation were performed according to the manufacturer’s instructions.

### Direct RNA sequencing and data processing

PolyA+RNAs were enriched from total RNAs using Dynabeads mRNA Purification Kit (Invitrogen). A total of 1,000 ng of polyA+RNAs were subjected to direct RNA sequencing library preparation using an SQK-RNA002 Kit [Oxford Nanopore Technologies (ONT), Oxford, UK]. The optional reverse transcription step was included using SuperScript III Reverse Transcriptase (Invitrogen). Sequencing was performed on the MinION platform using R9.4.1 flow cells (ONT).

Reads were basecalled using the Dorado workflow (v0.5.0) under the rna002_70bps_hac@v3 model (https://github.com/nanoporetech/dorado). The resulting FASTQ files were aligned to the TGEV genome (GenBank accession number: NC_038861.1) and *Sus scrofa* transcriptome (GenBank accession number: NC_010443.5) using Minimap2 v2.17 with parameter settings ‘-ax map-ont’ [30]. Mapping results were stored in SAM files, which were subsequently converted into bam files and sorted and indexed using SAMtools v1.17 [31]. Qualification control information was extracted using a custom Python script based on the result from SeqKit v2.9.0 (seqkit stats -a<basecalled fastq file>) and Giraffe v0.2.3 (giraffe estimate --input <basecalled fastq file>) [32,33]. Reads mapped to the host in the sample of healthy ST cells without infection will be referred to as ST-mock, in the sample of ST cells infected with TGEV-wt will be referred to as ST-wt and in the samples infected with TGEV-∆7 will be referred to as ST-∆7. Reads mapped to TGEV will be referred to as TGEV-wt and TGEV-∆7 according to the type of virus infected.

### Current feature showcase with nanoCEM

nanoCEM (version 0.0.6.3) was used to display Nanopore sequencing current at the site level [34]. All raw data were transformed into the blow5 format by blue-crab v0.30 (https://github.com/Psy-Fer/blue-crab) [35]. We selected the f5c_ev mode with the options ‘--norm’, ‘--rna’ and ‘--pore r9’.

### Detections of m6A sites

m6Anet (v1.1.1) relies on the eventalign module in Nanopolish (v0.14.0) to assign raw current signals to each nucleotide [23]. The signal features, including mean, standard deviation and signal length, were used as input to a multiple instance learning-based neural network model. We incorporated mod_ratio values computed as the m6A level for each DRACH in our analysis. Using all sites reported by m6Anet after quality control, we computed the global m6A proportion as the read-depth–weighted mean of per-site modification ratios:

 $p=\frac{\sum_{i}n_{i}r_{i}}{\sum_{i}n_{i}},$

where (r_i) denotes the m6Anet detected modification ratio at site (i) and (n_i) denotes the corresponding site coverage (read depth). Using all sites reported by m6Anet after QC, we aggregated reads within each condition into methylated and unmethylated counts and performed pairwise comparisons of global m6A proportions using Pearson’s chi-square test (2×2 contingency tables). *P*-values from the three pairwise comparisons were adjusted using the Benjamini–Hochberg procedure to control the false discovery rate (BH-FDR).

For the comparative methods, we applied a Python script to merge the results from Tombo and xPore (https://github.com/lrslab/Scripts_merge_DRS_methods) [20].

Tombo_com v1.5.1 is the canonical sample comparison method provided by Tombo. It offers two ways for modified base detection, ‘model_sample_compare’ and ‘level_sample_compare’. The latter approach was adopted in the present study. The ‘--store-p-value’ option was applied to save *P*-values for subsequent analysis. The ‘text_output’ option was employed to extract *P*-value and difference statistics.
xPore v2.1 employs the outputs derived from the Nanopolish eventalign module for all samples to generate a configuration file. The prediction of modified positions was determined by *P*-value and differential modification rate from the statistical test results.

#### Cutoff selection

We randomly split the reads from the uninfected control sample (ST-mock), which is biologically homogeneous with respect to infection status, into two technical subsamples. For each m6A site, we calculated the m6A proportion in each subsample and then computed the between-subsample difference (Δm6A proportion). Because the two subsamples originate from the same sample, the resulting Δm6A distribution represents a ‘null’ distribution of technical variance (i.e. differences expected in the absence of any true biological change).

We then used a two-tailed 99% confidence interval on this null distribution to establish symmetric comparative cutoffs. In this study, the 0.5th and 99.5th percentiles corresponded to ±0.330, meaning that 99% of site-wise differences observed between two technical splits fall within the range of −0.330 to +0.330. Accordingly, we adopted ±0.330 as the cutoff for calling dynamic m6A changes: only sites with an absolute difference in m6A proportion (|Δm6A|) greater than 0.330 were considered significantly changed.

Importantly, this approach directly anchors the threshold to the dataset’s own measurement uncertainty (including read-sampling effects and other technical fluctuations) rather than relying on an arbitrary fixed value. Under this criterion, an observed change exceeding ±0.330 has a <1% probability of arising from technical noise within a homogeneous sample, providing a conservative control of false positives. Therefore, we believe this represents a highly stringent and robust standard for identifying the most confidently and substantially altered m6A sites, ensuring that downstream interpretations focus on changes that clearly exceed background variability.

### DEG and DEI comparison analysis

A gene was classified as a differentially expressed gene (DEG) between tissues when meeting two criteria: (1) transcript abundance (measured in reads per million) demonstrated over two-fold change (|log_2_FoldChange|>1), (2) the false discovery rate (FDR) <0.05. Statistical analysis was performed using the edgeR package (version 3.30) in R, with FDR calculations conducted through Benjamini–Hochberg correction following quantile normalization of read counts [36]. A single gene can form different isoforms during transcription through alternative splicing, which is an essential post-transcriptional mechanism [37]. A transcript isoform was classified as a differentially expressed isoform (DEI) between two tissues if the alteration in its relative abundance (calculated as the percentage of its read count relative to all transcripts within the same genetic locus) exceeded 20% [38]. We use TrackCluster (version 0.1.7) to identify isoforms [39]. Then, the ClusterProfiler package was used to deal with Gene Ontology (GO) enrichment analysis to explore the enrichment of certain functions or features in the DEG and DEI gene sets, respectively [40]. For all GO enrichment analyses, a stringent significance cutoff of p.adjust <0.01 was applied.

### Viral gRNA and sgRNA expression

After virus entry into cells, genomic RNA will serve as the initial template to combine a nested set of sgRNAs with the same 5′ UTR leading [41]. SgRNAs always start from a certain ORF of the virus and completely contain the remaining sequence of the viral genomic RNA until the 3′ polyA tail. But only the ORF closest to 5′ UTR would be translated, whether genomic RNA or subgenomic RNAs [41]. Therefore, we divided the mRNA mapped to the viral genome into gRNA and different sgRNAs according to the viral ORF to which the mRNA 5′ belongs. Considering that nanopore sequencing starts from the 3′ end, the 5′ end sequence may be degraded during the process, resulting in an incomplete ORF. After deleting the polyA tail, we only require counting from the 3′ end of the reads and at least one base after that belongs to the corresponding ORF. The production of ORF1b depends on the ribosomal frameshift that occurs after the production of ORF1a, so ORF1b does not generate new sgRNAs [42]. Instead, it forms a larger protein together with ORF1a, so it is still expressed using gRNA, as when ORF1a is expressed alone. In addition, it should be emphasized that the mutation at the front end of the TGEV gene 7 will only cause the protein encoded by gene 7 to fail to be translated and will not affect the production of the sgRNA of gene 7. After counts per million (CPM), we used the relative expression of ORFs to compare the effect of gene 7 abrogation on the expression of viral ORFs.

### PolyA length estimate

Nanopolish (version 10.2) was used to calculate polyA length of each read with the command ‘nanopolish polya’ (https://github.com/jts/nanopolish) [43]. The length of the polyA was inferred from the dwell time of the raw ion signal at the unaligned part of the 3′ end of the reads.

We only retained reads with polyA length ≤250 nt for further analysis. We first performed kernel density estimation on the data of gRNA and each sgRNA to identify peaks in the distribution of polyA tail length. The local maximum (peak) of the density curve was detected as a point with a higher density than the adjacent points. This process was implemented in R using the density() function (adjust=1.2). To reduce false positives caused by small fluctuations, only peaks with a signal-to-noise ratio greater than 1 were considered. To quantify the uncertainty of peak-based inference in the absence of technical replicates, we assessed the stability of the dominant peak location by non-parametric bootstrap resampling. Specifically, we restricted the analysis to Nanopolish polyA calls with qc_tag=PASS and polyA length ≤250 nt. For each group×read_type (gRNA and each sgRNA), we performed bootstrap re-sampling with replacement (1,000 iterations) at the read level. In each bootstrap iteration, we re-estimated the polyA length distribution by kernel density estimation (R density(), adjust=1.2) and re-identified the dominant mode as the local maximum with the largest density among peaks passing the signal-to-noise threshold. The 2.5th and 97.5th percentiles of the resulting bootstrap peak positions were used as the 95% confidence interval (CI) for the peak location. We further computed Δpeak values between conditions (and, where indicated, relative to ORF1) and reported the corresponding uncertainty using the bootstrap-derived peak distributions.

## Results

### Direct RNA sequencing obtained enough reads for ST cell and TGEV transcriptome analysis

**Fig. 1.**
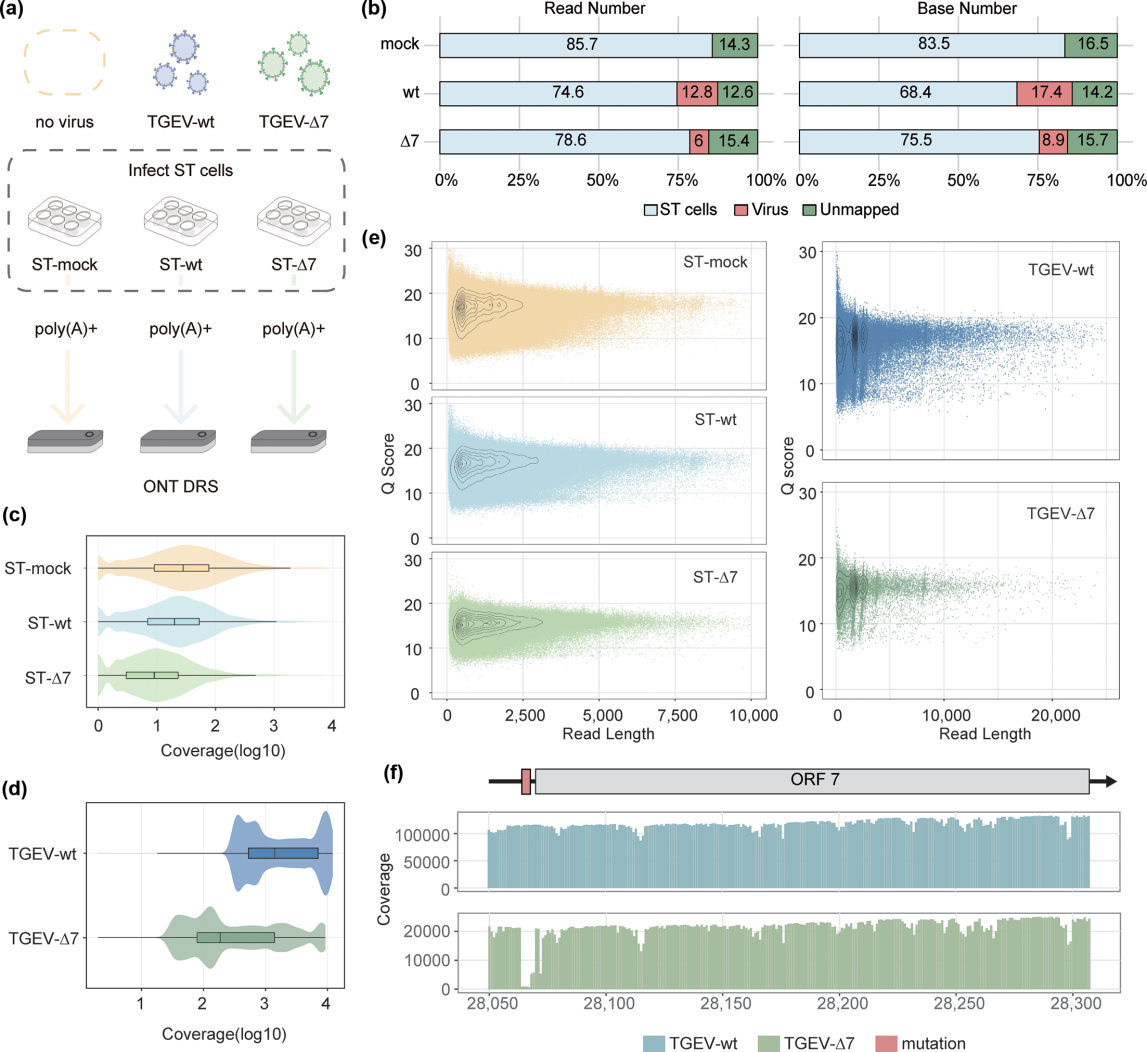
Raw read characterization and mapped reads analysis. (**a**) Comparative experimental design and sequencing process of each group. (**b**) The proportion of host reads and viral reads from the three sequenced samples. (**c**) Gene coverage of reads mapped to the swine genome. (**d**) Genes’ coverage of reads mapped to the viral genome. (**e**) Distribution of *Q* scores for different read lengths. As the read length increases, the *Q* scores of both viruses remain at a high level, with no significant downward trend. (**f**) Coverage bar plot around the TGEV gene 7 showed a frameshift mutation caused by a three-base deletion at the front end.

ST cells were infected at MOI 1 with TGEV or TGEV-∆7. At 16 h post-infection, when the virus reached its highest replication titre, polyadenylated RNAs were isolated from infected cells and sequenced via ONT direct RNA sequencing (Fig. S1, available in the online Supplementary Material). The healthy ST cell without infection was also collected and sequenced as the mock (Fig. 1a).

After basecalling, we obtained 1,918,474, 1,248,609 and 486,170 reads from mock, TGEV-wt infected sample and TGEV-∆7 infected sample, respectively. Over 85% of reads from each sample could be mapped to the swine genome or TGEV genome, with no TGEV-mapped reads in mock, confirming the absence of pre-existing virus contamination (Fig. 1b, Table S1). Additionally, viral RNA abundance differed markedly between infections, with the proportion of TGEV-wt being two times higher than TGEV-∆7 (12.8% vs. 6% in read number or 17.4% vs. 8.9% in base number, Fig. 1b), confirming TGEV gene 7 was a key virulence factor. Host-derived mRNA reads were hereafter designed as ST-mock (uninfected), ST-wt (TGEV-wt-infected) and ST-∆7 (TGEV-∆7-infected), while viral reads were labelled according to their respective strains (TGEV-wt or TGEV-∆7).

**Fig. 2.**
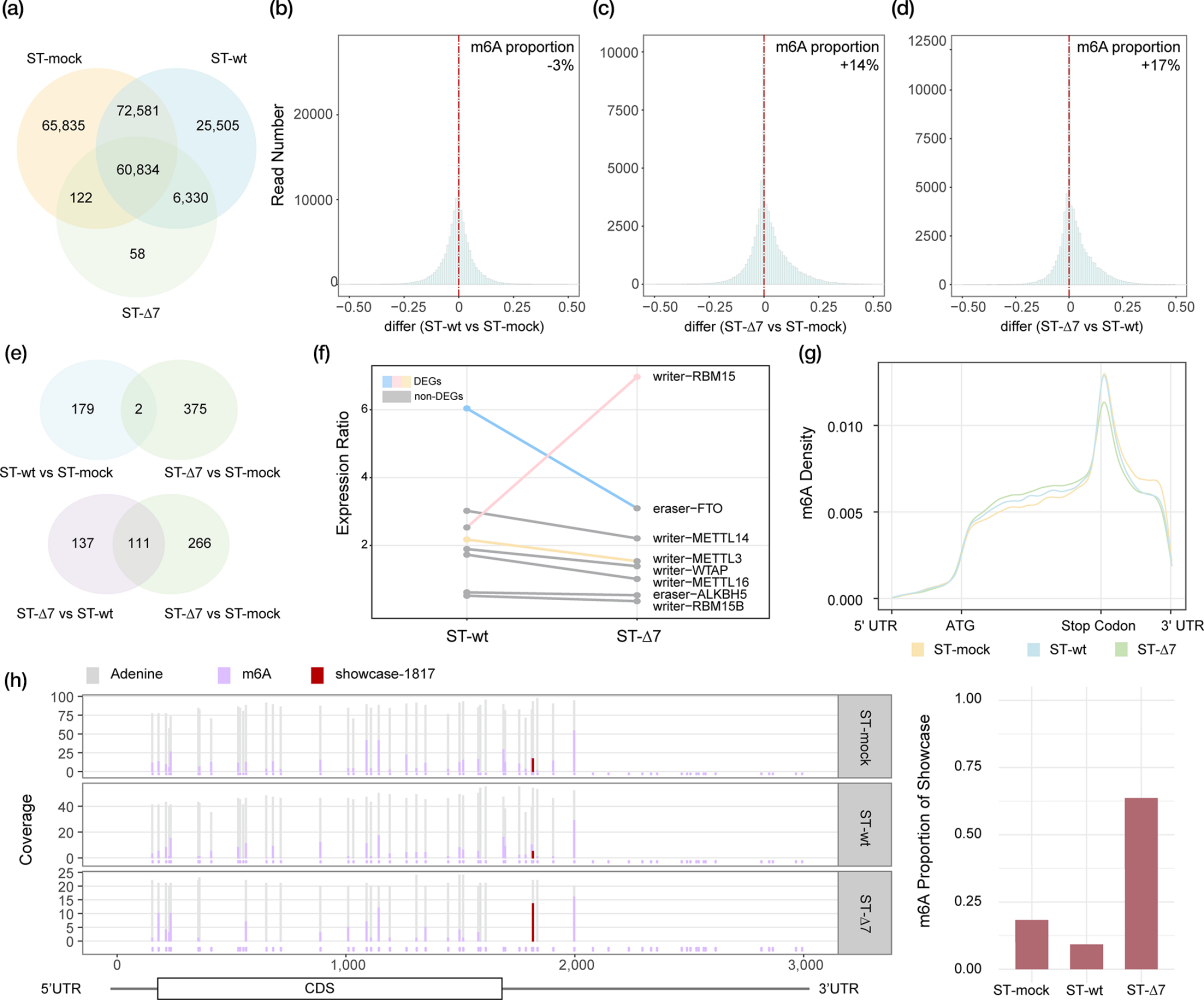
Overall analysis of m6A modification in ST cells. (**a**) m6A sites (DRACH sequence) of host (ST cell) detected by m6Anet. The overall difference in m6A levels between ST-wt and ST-mock (**b**), ST-∆7 and ST-mock (**c**) and ST-∆7 and ST-wt (**d**). (**e**) The overlapping of significantly changed m6A sites between pairwise comparisons. Here, we require not only the same site, but also the same modification changes at the site. (**f**) Expression ratio of genes regulating m6A levels compared with ST-mock. Coloured lines indicate that the gene is a statistically differentially expressed gene in at least one group, and the points of that group are also coloured; otherwise, they are grey. (**g**) m6A site density in ST cell mRNA. (**h**) Methylation proportion of each m6A site around gene SNX1. The dark red site (located at base 1817 of the gene) is used as a showcase to show the changes in the proportion of m6A in different samples.

Host transcriptome sequencing achieved comprehensive genomic representation, with over 95% of annotated swine genes detected across samples (Fig. 1c). Viral sites showed robust coverage, maintaining a minimum median depth of 50×, which is enough for reliable direct RNA sequencing-based RNA modification analysis (Fig. 1d) [23]. All groups demonstrated consistent sequencing quality, with median *Q* score of 16.65 (ST-mock), 16.47 (ST-wt) and 15.28 (ST-∆7) for host reads and 16.51 (TGEV-wt) and 15.26 (TGEV-∆7) for viral reads. In addition, the GC content of TGEV-wt in the viral sequencing results was 38.2 mol%, and the GC content of TGEV-∆7 was 38.1 mol%, which is consistent with the overall low GC content characteristics of the *Coronaviridae* family [44] (Table 1). Viral read length distributions revealed four peaks at 1,500 nt, 2,500 nt, 3,800 nt and 8,000 nt (Figs. 1e, S2 and Table S2). These peaks were assigned to specific viral transcripts by correlating their lengths with the expected sizes of the TGEV gRNA and the nested set of sgRNAs that encode the nucleoprotein, envelope protein, non-structural protein 3 and spike protein, respectively. We checked coverage per site and found that nanopore direct RNA sequencing can capture the three-base mutation in front of gene 7 as described in the previous article, determining the correct abrogation of the TGEV gene 7 in TGEV-∆7 sample (Fig. 1f).

**Table 1.** Raw data feature based on Dorado basecalling

	No. of reads	Average length	N50	Median *Q* score	GC (mol%)
**ST-mock**	1,643,837	1,194.7	1,609	16.65	50.8
**ST-wt**	931,761	1,412.1	1,923	16.47	49.2
**ST-∆7**	382,124	1,409.9	1,861	15.28	49.5
**TGEV-wt**	159,496	1,991.6	2,574	16.51	38.2
**TGEV-∆7**	29,227	2,041.2	2,544	15.26	38.1

Our nanopore direct RNA sequencing approach generated high-coverage, quality-controlled datasets across all experimental groups and confirmed the loss-of-function mutation of gene 7 in TGEV-∆7 virus while ensuring comparability between wild-type and mutant datasets. This foundational data quality enabled system-level analysis of transcriptomic and epitranscriptomic alterations and mechanistic dissection of TGEV gene 7’s function in modulating RNA processing.

### Inverse regulation of host m6A methylation landscapes between TGEV-wt and TGEV-∆7 infection

Around 230,000 DRACH sites were detected by m6Anet in swine transcriptomes. Across DRACH motifs (sites with ≥5 supporting reads), we quantified the overlap of m6A-positive sites among conditions (Fig. 2a). A total of 60,834 DRACH sites were shared by all three conditions (ST-mock ∩, ST-wt ∩ and ST-Δ7). Pairwise overlaps (including the three-way intersection) were 133,415 sites for ST-wt vs. ST-mock, 67,164 sites for ST-wt vs. ST-Δ7 and 60,956 sites for ST-mock vs. ST-Δ7. Condition-specific DRACH sites numbered 65,835 (ST-mock only), 25,505 (ST-wt only) and 58 (ST-Δ7 only).

The global m6A proportion was 0.1254 in ST-wt, 0.1289 in ST-mock and 0.1469 in ST-Δ7. Pairwise comparisons using a chi-square test on aggregated methylated vs. unmethylated read counts, with Benjamini–Hochberg correction for the three comparisons, showed that ST-Δ7 had a higher global m6A proportion than ST-mock (Δ*p*=+0.0180; BH-FDR *q*<2.2×10⁻¹⁶, here, Δp denotes the difference in global m6A proportion between the two conditions) and ST-wt (Δ*p*=+0.0216; BH-FDR *q*<2.2×10⁻¹⁶), while ST-wt was slightly lower than ST-mock (Δ*p*=−0.00354; BH-FDR *q*=1.52×10⁻¹⁹³). To identify the most significantly changed m6A sites, as described in the ‘Methods’ section, we randomly split the reads of the uninfected control sample (ST-mock) into two technical subsamples and constructed a ‘technical noise’ null distribution of the difference in their m6A proportions. We obtained a ±0.33 threshold using a two-sided 99% CI. Therefore, we defined only sites where |Δm6A|>0.33 (probability of <1% due to background noise within the same sample) as significant dynamic changes, thus ensuring that the screening results are highly rigorous and reliable.

During TGEV-wt infection, the overall proportion of m6A modifications in mRNA decreased (Fig. 2b). Compared to ST-mock (healthy, uninfected ST cells), 52,567 m6A sites in ST-wt were increased, while 62,495 sites showed reduced m6A levels. On average, the global m6A level decreased by ~3%. By applying a threshold of >0.330 to identify significantly changed sites, we found that the ST-wt sample had 67 sites with significantly higher m6A modification and 114 sites with significantly lower modification (Tables S3 and S4).

The ST cell infected with TGEV-∆7 showed a different trend of overall m6A modification change. When compared with ST-mock, we found that 31,303 m6A sites in ST-∆7 showed higher m6A modification proportion, while 23,204 sites were lower (Fig. 2c). The overall increase in m6A proportion after TGEV-∆7 infection was 14%. For the selection of significantly changed sites, 332 sites were significantly higher, and only 42 sites were significantly lower (Table S5).

When comparing the differentially infected ST cells, the difference in m6A proportion became more obvious. There were 36,786 m6A sites in ST-∆7 that had a higher m6A proportion, while 22,238 sites in ST-wt had a higher m6A proportion (Fig. 2d). The overall increase of ST cell m6A modification between TGEV-∆7 infection and TGEV-wt infection was 17%. For the significantly changed m6A sites, we detected 239 sites that had significantly higher modification, and 9 sites had significantly lower modification in ST-∆7 when compared to ST-wt (Table S6).

Analysis of significantly changed sites also showed that when using ST-mock as the background, the m6A modification of the two infection groups was very different. There were only two overlaps of sites with the same change trend in the two infected samples, and the similarity of changes caused by different infections was very low. Compared with ST-wt or ST-mock, 111 points of ST-∆7 changed in the same direction, indicating that deletion of TGEV gene 7 dominated the production of specific epigenetic responses (Fig. 2e). TGEV gene 7 had a strong regulatory effect on host m6A modification over infection.

To understand the mechanism behind the altered m6A landscapes, we next examined the expression of key regulatory enzymes. Consistent with our hypothesis that gene 7 modulates the m6A machinery. Refer to writer genes responsible for m6A methylation and eraser genes responsible for m6A demethylation that had been identified [13]. We examined the expression fold of writers and erasers in ST-wt and ST-∆7 by using the gene expression of ST-mock as the baseline. m6A modification regulators in ST-wt and ST-7 were activated to various degrees. Fat mass and obesity-associated gene (FTO, eraser) was significantly upregulated in ST-wt by two times higher than ST-∆7. In contrast, in ST-∆7, putative RNA-binding protein 15 (RBM 15, writer) was significantly upregulated by 2.6 times higher than ST-wt (Fig. 2f). The expression fold of the regulatory gene explains the observed decrease in methylation levels in ST-wt and increase in ST-∆7m6A.

Even though the m6A proportion changed greatly, the distribution of modification sites on CDS remained stable. All ST cells showed enrichment of m6A modification near the stop codon (Fig. 2g), which is consistent with the distribution of m6A modification in mammals [45]. Most of the measurable points near the stop codon were consistent with the trend that ST-∆7 was greater than ST-mock and ST-wt. For example, the m6A site close to the stop codon of the gene SNX1 showed a dramatic increase in ST-∆7 (Fig. 2h).

### TGEV-Δ7 exhibited enhanced viral RNA m6A methylation compared to TGEV-wt

Viral RNA modification differences between TGEV-wt and TGEV-∆7 were observed using m6Anet and a pipeline integrating Tombo and xPore as a secondary verification method (Fig. S5). Comprehensive viral m6A profiling revealed systematic hyper-modification in TGEV-∆7.

**Fig. 3.**
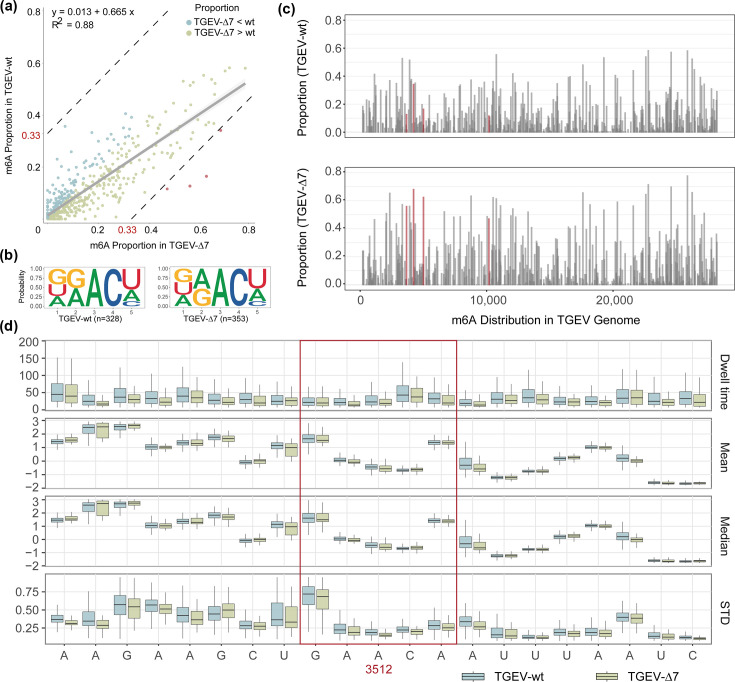
Comparison of the m6A site in viral RNA detected by m6Anet and the intersection of xPore and Tombo. (**a**) RNA m6A modification proportions detected by m6Anet. The correlation of the m6A proportions between TGEV-wt and TGEV-7 was examined by linear regression. The significantly different m6A sites are coloured in red. (**b**) Sequence logos of the 5-mer sequence context (m6Anet-reported k-mers, to maintain consistency with the current signal, all T were replaced with U) centred on the predicted modified adenosine (position 3) are shown for TGEV-wt and TGEV-Δ7, illustrating enrichment of the canonical DRACH motif (D=A/G/U, R=A/G, H=A/C/U). (**c**) Genomic distribution of m6Anet detected modification sites in the viral genome. The four significantly differed m6A sites are highlighted in red. (**d**) The ONT raw current changes of the m6A site at position 3512. The red frame highlighted was the DRACH site (GAACA).

In total, 596 m6A sites with DRACH motif could be profiled using m6Anet (Table S7). Among them, 336 sites showed higher modification proportion in TGEV-∆7, when compared with TGEV-wt. Only 201 sites displayed a reversed trend (Fig. 3a). The summed proportion from all m6A sites in TGEV-∆7 was 32% higher than that in TGEV-wt (Fig. 3a). The heatmap of m6A proportions at all analysed sites further illustrates the increase in TGEV-Δ7 across the entire genome (Fig. S7). The 5-mer sequence logos centred on the modified adenosine were highly similar between TGEV-wt (*n*=328) and TGEV-Δ7 (*n*=353), and both conformed to the canonical DRACH consensus, indicating that the Δ7 deletion does not measurably alter the underlying m6A motif preference (Fig. 3b).

Although there was no evidence of motif spatial clustering in the viral genome, we identified the four most significantly different sites with a threshold of m6A proportion difference above 0.330 (Fig. 3a). All four sites were located in ORF1a (Fig. 3c) and exhibited elevated m6A in TGEV-∆7 (Fig. 3a, c). The m6A modification at the DRACH motif would likely lead to a decrease in raw current signal compared to the canonical A [46]. This principle allows the detection of modification proportions. To validate the increased m6A proportion predicted by m6Anet at specific sites in TGEV-Δ7, we examined the raw current traces using nanoCEM. For example, at the DRACH site (GAACA) at genomic position 3512 – one of the four sites with the most significant increase – we observed a clear decrease in the median current level in TGEV-Δ7 reads compared to TGEV-wt reads, and the time of sequencing (Dwell time) of nearby bases was higher (Figs 3d, S8 and S9). This consistent current reduction provides orthogonal support for the increased m6A modification stoichiometry at this locus in the gene 7-deleted virus (Fig. 3d).

The results of xPore and Tombo were combined to obtain 344 sites with potential modification differences (Fig. S5). These 344 sites were further combined with the results of m6Anet, and only 23 common sites were found. According to the results of m6Anet, 16 sites showed a higher proportion of m6A modification in TGEV-∆7 than in TGEV-wt (Table S6).

Generally speaking, TGEV-wt and TGEV-Δ7 also showed trends in m6A changes similar to those of the host (Figs2d  3a, b). In the comparison of the infected groups, we found that the m6A in both host and viruses increased in the infected group lacking the TGEV gene 7. Suggesting that TGEV employed gene 7 to avoid the host m6A modification, as a deletion of gene 7 would reverse the effect.

### Conserved transcriptomic profiles with enhanced host antiviral resistance in TGEV-∆7 infection

**Fig. 4.**
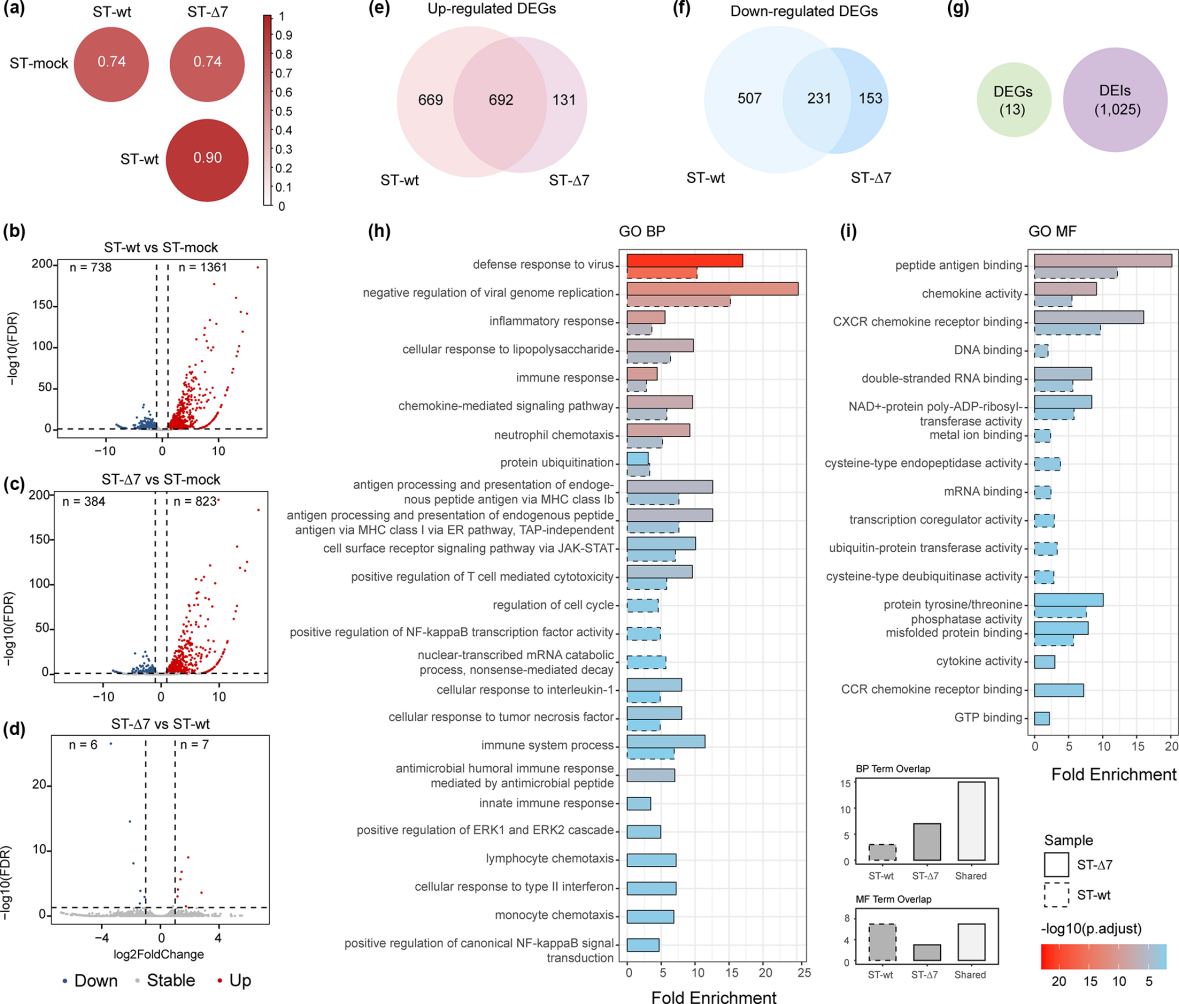
Comparative analysis of host DEGs and DEIs. (**a**) Spearman correlation analysis of transcriptome response between healthy ST cells (ST-mock), ST cells infected with TGEV-wt (ST-wt) and ST cells infected with TGEV-∆7 (ST-∆7). Volcano plots of DEGs (|log_2_FC| ≥1, FDR §amp;lt; 0.05) gained comparison between ST-wt and ST-mock (**b**), ST-∆7 and ST-mock (**c**) and ST-∆7 and ST-wt (**d**). The DEGs obtained by comparing the two different infection groups with the mock group overlapped, and both upregulated DEGs (**e**) and down-regulated DEGs (**f**) showed a significant intersection. (**g**) Overlap of DEGs and DEIs between ST -wt and ST-∆7. GO enrichment analysis of upregulated DEGs with p.adjust <0.01 for (**h**) biological process and (**i**) molecular function categories. The dashed boxes represent terms enriched in ST-wt, and the solid boxes represent terms enriched in ST-∆7. The colour of the boxes corresponds to the statistical significance of the enrichment, represented as -log10(p.adjust), as shown in the vertical scale bar. The size of the dots represents the fold enrichment for each term, as indicated by the legend on the right. In the overlap diagrams (below H and I), the dashed grey boxes represent the number of terms unique to ST-wt, the solid grey boxes represent the number of terms unique to ST-∆7 and the solid white boxes represent terms common to both samples within the same category.

We used TrackCluster to quantify the host transcriptome. Stringent thresholds (genes, total counts >15; isoforms, total counts>6) were applied to confirm the expression of genes and isoforms. Correlation analysis revealed high similarity between ST-wt and ST-∆7 (Fig. 4a).

DEG analysis showed TGEV-wt infection induced more pronounced changes. When compared with ST-mock, ST-wt showed a total of 1,361 significant upregulated genes and 738 significant down-regulated genes (Fig. 4b). While ST-∆7 showed only 823 upregulated genes and 384 down-regulated genes (Fig. 4c). Direct comparison between ST-∆7 and ST-wt showed minimal difference with only 13 DEGs (Fig. 4d). Although ST-wt vs. ST-mock showed more DEGs compared with ST-∆7 vs. ST-mock, more than half of the DEGs obtained in the two comparisons were the same (Fig. 4e, f). Notably, while gene-level changes were limited, we observed numerous DEIs in ST-wt vs. ST-Δ7, indicating isoform-level regulation could be independent from gene level (Fig. 4g).

Having established that gene 7 deletion leads to a hyper-methylated state, we sought to determine its functional consequences for the host response. Supporting our hypothesis that increased m6A potentiates immunity. We performed GO enrichment analysis on the DEGs using a stringent significance cutoff (p.adjust <0.01). This analysis revealed that the upregulated DEGs in both ST-wt and ST-Δ7 infections were significantly enriched in highly similar ‘biological process’ and ‘molecular function’ categories, which were dominated by themes of antiviral defence and inflammatory signalling (Fig. 4h, i, Table S8). Only the down-regulated DEGs from ST-wt showed two significant enrichments (mitochondrion and mitochondrial outer membrane) for the ‘cellular component’ category under this cutoff (Table S9).

Compared with ST-mock, most DEGs enriched in biological processes were upregulated, and there was striking convergence: ST-wt was enriched in 18 pathways, ST-∆7 in 22, with 15 pathways shared (Fig. 4h, Table S8). Both ST-wt and ST-∆7 showed strong antiviral immunity and inflammation themes, but the enrichment fold and significance of ST-∆7 were generally higher (for example, ST-∆7’s ‘defence response to virus’ fold enrichment=17.05 vs. ST-wt’s 10.29), and ST-∆7 contained more chemotaxis-related terms (such as ‘neutrophil chemotaxis’). This suggests that the immune response of ST-∆7 is more intense and may involve a wider range of cell types (such as lymphocyte chemotaxis). The only enriched term among the down-regulated DEGs involved mitochondrial cytochrome c oxidase assembly, indicating that mitochondrial energy metabolism or oxidative phosphorylation may be inhibited.

In molecular function (MF), the significantly enriched terms were all concentrated in upregulated DEGs and related to viral defence and inflammatory signals (Fig. 4i, Table S10). In general, ST-wt vs. ST-mock was enriched in 14 pathways, and ST-∆7 vs. ST-mock was enriched in 10 pathways, of which 7 pathways were shared by the two groups. Both samples were enriched for antigen presentation (SLA family) and chemokine-mediated immune cell recruitment, indicating similar basic immune activation mechanisms. ST-∆7 had enhanced antiviral recognition: the enrichment fold of dsRNA binding function increased by 50%, and the OASL gene was only expressed in ST-∆7 but not ST-wt. Inflammatory signals were also expanded, with new cytokine activity (IFN/IL6/TNF) and CCR chemotaxis. For ST-∆7, the *P*-values of the same functions were lower, and the enrichment folds were higher. Analysis of MF also indicated that TGEV-∆7 could lead to a more active immune response, involving a wider range of inflammatory mediators (cytokines) and more efficient viral RNA recognition mechanisms (dsRNA binding).

Overall, gene enrichment analysis showed that TGEV-Δ7 infection triggered a stronger host immune response than TGEV-wt infection. This suggests that gene 7 of TGEV has the function of suppressing antiviral immunity. Consistent with this, TGEV-Δ7-infected cells consistently showed significantly higher enrichment of upregulated DEGs in antiviral and inflammatory pathways compared with TGEV-wt infection.

### Sharp decline in viral overall RNA replication with stable relative gene expression

**Fig. 5.**
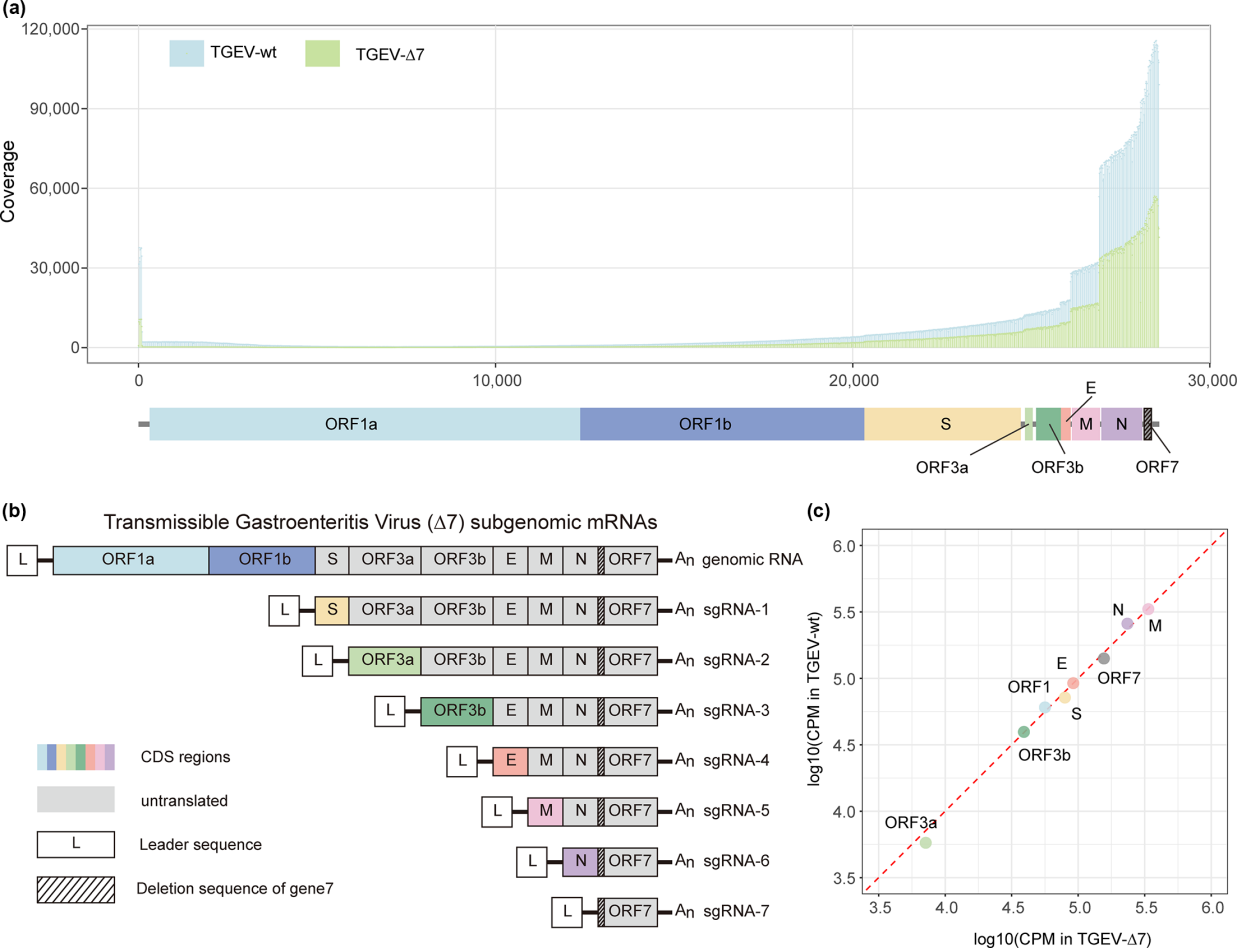
Relative expression of viral gRNA and sgRNAs. (**a**) Coverage of each site of sequencing data aligned to the TGEV reference. Blue bars represent TGEV-wt (above), and green bars represent TGEV-∆7 (below). The *x*-axis is the site location in the virus genome, and the *y*-axis is site coverage after normalizing (coverage/all reads×10^6^). (**b**) Genomic and subgenomic RNAs were produced during TGEV infection. Three bases were deleted at the beginning of the sequence of ORF7, indicated by the dotted area, which prevents gene 7 from being successfully translated. (**c**) Correlation of genomic and subgenomic RNA expression between wild-type TGEV (TGEV-wt) and recombinant TGEV, which abrogated gene 7 (TGEV-∆7). Each point represents the log10(CPM) value of one gRNA or sgRNA.

The number of viral reads was normalized by CPM and aligned with the annotated viral genome to obtain the distribution map of virus reads on the genome. The step-like distribution is consistent with the characteristics of viruses using gRNA and sgRNAs for transcription (Fig. 5a, b). We noticed that while the overall trend was similar, the expression level of TGEV-∆7 could only reach half of that of TGEV-wt (Fig. 5a). Viral mRNAs could be grouped based on the characteristics of TGEV gRNA and sgRNAs (Fig. 5b). As gRNA and sgRNAs only translate the gene closest to the 5′ UTR during translation, we grouped reads according to which ORF the 5′ end base belongs to after mapping to the viral genome (Fig. 5b). We found that the relative fold change of gene expression between TGEV-∆7 and TGEV-wt was all close to 1, and no difference could be observed (Fig. 5c). The abrogation of the TGEV gene 7 would not alter the expression ratio of viral genes.

### Deletion of gene 7 leads to a significant extension of the viral polyA tail during infection

We used genes with average CPM≥5 to carry out the polyA tail analysis. Firstly, all reads of genes that pass the CPM filter were used for analysis. The median polyA length of ST-mock was ~105. ST-wt showed a significant extension of ~3 nt, while the change in ST-∆7 polyA length was not statistically significant (Fig. S11A). TGEV-wt infection would prolong the mean length of polyA tail, but abrogation of TGEV gene 7 alleviated this situation.

Recent studies have shown that short-tailed transcripts tend to be highly expressed [47]. To examine if the gene expression had an impact on polyA length, we separated the reads from each gene to calculate gene-level polyA length. We found that the median polyA length of ST-mock was ~128 nt, and the median polyA lengths of ST-wt and ST-∆7 were both ~125 nt (Fig. S11B, Table S11). The polyA tail distribution results of ST-wt and ST-∆7 are basically the same (Fig. S10). Generally, the polyA tail length of the host cell showed a slight decrease after infection, but TGEV gene 7 did not bring additional effects.

We also counted the polyA length from viral transcripts. Measured against SARS-CoV-2, TGEV-wt had a relatively long polyA tail, with a median length of 64 nt [48]. An abrogation of the TGEV gene 7 was associated with a modest but statistically significant increase in polyA tail length (≈7 nt on average) across viral transcripts; the functional relevance of this shift remains to be determined (Fig. S11C). For gRNA and sgRNAs, in TGEV-wt, there were two polyA tail populations in sgRNAs used to translate ORF3b, M and N. The minor peak was around 33~37 nt, while the major peak was around 60~70 nt. However, the polyA length distribution for sgRNA-7 (translate ORF7) was different from all other sgRNAs, with the highest peak at only 30 nt (Fig. S11D).

When compared with TGEV-wt, TGEV-∆7 exhibited an additional major peak in sgRNA-7 at 73 nt, while the minor peaks of sgRNA-3 and −5 disappeared (Fig. S11D, Table S12). Compared with TGEV-wt, most polyA tails of TGEV-∆7 gRNA and sgRNAs increased by 3~10 nt except for sgRNA-1 and sgRNA-2 (Fig. S11E, Table S13). To evaluate whether the observed peak shifts exceeded the uncertainty of Nanopolish-based polyA estimates and our peak-calling procedure, we quantified the stability of the dominant peak position using non-parametric bootstrap re-sampling within each condition and transcript class (see the ‘Methods’ section). Briefly, for each gRNA/sgRNA distribution, we re-sampled reads with replacement (1,000 iterations), re-estimated the kernel density curve and re-identified the dominant mode to obtain a 95% CI for the peak location. For most transcript classes, the peak-location CIs were narrow, whereas the inferred shifts between TGEV-wt and TGEV-∆7 for several sgRNAs were markedly larger than the bootstrap uncertainty, supporting that these differences are unlikely to be explained by sampling fluctuation or instability of KDE peak-picking. In contrast, sgRNA-7 in TGEV-∆7 showed a comparatively wide CI and an apparently unstable/multi-modal distribution; therefore, we interpret sgRNA-7 cautiously and do not rely solely on a single-mode summary for this class (Table S14).

Notably, the overall polyA length seemed to align with the length of gRNA and sgRNAs (Fig. S11E, Spearman correlation R of TGEV-wt=0.95, TGEV-∆7=1). This trend led to a huge difference in the polyA length between gRNA and sgRNAs (Table S13). For example, the difference between TGEV-wt gRNA and sgRNA-7 reached 32.5 nt.

The different trend of host and viral polyA tail between TGEV-wt- and TGEV-∆7-infected samples indicated that TGEV had a polyA tail regulation mechanism independent from the host. The coronavirus protein translation efficiency was positively related to polyA length [49]. The overall polyA tail extension in TGEV-∆7 would likely alleviate the adverse effects of the sharp decrease in TGEV-∆7 RNA replication by improving the efficiency of translation.

## Discussion

By using nanopore direct RNA sequencing, we demonstrated a link between TGEV non-structural protein gene 7 and m6A modifications. Collectively, our data support a gene 7-dependent model in which TGEV remodels the host m6A landscape by differentially tuning key components of the m6A machinery. Specifically, infection with TGEV-wt significantly upregulates the m6A demethylase FTO, and this change is associated with a ~3% relative decrease in global host m6A levels as inferred from Nanopore direct RNA sequencing. By contrast, infection with TGEV-Δ7 robustly induces RBM15, a writer complex-associated regulator, and this induction coincides with a ~14% relative increase in global host m6A levels based on the same Nanopore-derived estimates. Together, these results suggest that gene 7 may bias infection towards an m6A-lowering state (potentially via FTO upregulation), whereas its ablation shifts the balance towards increased m6A deposition (potentially via RBM15 induction), with downstream consequences for host antiviral programmes and viral RNA fate. GO analysis of DEGs revealed that loss of TGEV gene 7 enhanced antiviral and chemokine pathways, and the resultant attenuation was further reflected by a decline in viral RNA replication. Nanopore dRNA-seq also provides additional layers of information, such as polyA tail length distributions, which we report as descriptive but informative readouts of the broader host–virus interaction landscape. While these features may reflect downstream consequences of gene 7-dependent infection biology, they are presented here mainly to generate mechanistic hypotheses for future targeted validation.

While this high-throughput study offered innovative insights, we acknowledged several limitations. First, the absence of biological replicates and the comparatively low sequencing depth for TGEV-∆7-infected ST cells could have introduced bias. Furthermore, due to the discontinuation of the SQK-RNA002 kit (ONT’s protocol for full-length polyadenylated RNA) and its replacement by SQK-RNA004, we are unable to generate additional direct RNA sequencing datasets that are strictly comparable to the original data. Based on our benchmarking, RNA004 and RNA002 produce markedly different outputs, and therefore, RNA004 is not appropriate for performing supplementary experiments intended to replicate RNA002-derived differential expression or m6A analyses. Although we were unable to perform a true retest under identical conditions, random subsampling of reads from each group showed high consistency with the full dataset for both DEG detection and Nanopore-inferred m6A analyses, supporting the internal reproducibility of the sequencing results (Figs S3 and S6, Table S15). To further assess the robustness of our findings in the absence of additional strictly comparable DRS replicates, we performed an internal reproducibility analysis by randomly subsampling the original Nanopore DRS reads and repeating the full downstream workflow. We compared (i) differential expression results derived from the full vs. subsampled datasets and (ii) m6A-related signals inferred by m6Anet between the two analyses. The differential expression results were highly concordant, with >91% overlap of DEGs between full and subsampled analyses for both ST-wt and ST-Δ7, and the m6Anet-based m6A profiles showed similarly strong consistency. Together, these results indicate that our main transcriptomic and epitranscriptomic conclusions are internally robust to sequencing depth and stochastic read sampling. Second, without *in vitro-*transcribed, unmodified RNA as a negative control, we were unable to validate the accuracy of the results of m6Anet-identified viral m6A sites. Finally, our xPore-Tombo pipeline could not quantify absolute changes in modification stoichiometry, and its sensitivity remained uncertain without a corresponding unmodified reference.

We proposed that the diminished replication observed in TGEV-∆7 was associated with the upregulation of m6A levels. Supporting this notion, suppressing host FTO, the m6A eraser, has been shown to impair replication of several RNA viruses [15]. Accordingly, the loss of gene 7 likely reduced viral replication indirectly by tilting the host epitranscriptomic balance toward increased m6A. The accumulation of m6A modification could also modulate innate immunity, as documented in SARS-CoV-2 infection [48]. Importantly, although mechanistic evidence remains more limited in *Alphacoronavirus* systems, related observations have been described in PEDV and HCoV-229E, supporting the possibility that epitranscriptomic regulation represents a shared layer of host–virus interaction across *Coronaviridae* [17,50]. Our data indicated that gene 7 acted as an immune-evasion factor by simultaneously down-regulating the writer RBM15 and upregulating the eraser FTO, thereby preventing excessive m6A on both host and viral RNAs and safeguarding viral replication. This mechanism may explain why TGEV infection lowered host m6A, whereas several beta coronavirus infections have been reported to elevate it [7], highlighting both potential conservation (RNA-centric regulation) and divergence (directionality of m6A changes) among coronavirus lineages.

In this study, deleting the TGEV gene 7 was associated with an ~7 nt extension of the 3′ polyA tail. Because polyA length is a key determinant of RNA turnover, this shift may reflect increased stability of viral RNAs in the absence of gene 7. Longer polyA tails can enhance recruitment of polyA-binding protein (PABP) and related host RNA-binding factors, which typically protect transcripts from 3′–5′ decay and can secondarily promote translation. Thus, polyA tail extension in the gene 7 deletion virus may represent a compensatory adaptation that helps maintain viral RNA abundance when gene 7-dependent functions are lost.

The observed polyA change also points to altered virus–host interactions. Remodelling of the PABP/translation machinery and cytoplasmic RNA decay pathways is tightly linked to innate immune control of viral infection. A longer polyA tail could shift the balance between stabilization vs. clearance of viral RNA and thereby modulate the magnitude and kinetics of RNA-sensing pathways (e.g. RIG-I/MDA5-driven responses) indirectly through changes in RNA availability and ribonucleoprotein composition. Collectively, our results suggest that TGEV gene 7 influences 3′-end biology and host-factor engagement, with measurable consequences for viral RNA stability and the host antiviral landscape.

Contrary to the negative correlation between polyA length and RNA modification reported previously [48]. Our study showed a direct positive correlation between polyA tail extension and increased m6A levels in TGEV-∆7. This discrepancy may stem from fundamental differences in the predominant RNA modification motifs examined. The SARS-CoV-2 study focused on the AAGAA motif [48], whereas our analysis targeted the canonical DRACH consensus for identifying m6A sites. Taken together, these observations broaden the framework for antiviral strategies that target RNA stability or epitranscriptomic pathways. Future research should dissect motif-specific links between m6A and polyA regulation and evaluate the universality of these mechanisms across viral taxa.

Beyond molecular insights, our research has significant translational implications. TGEV remains a major threat to the global swine industry, causing severe economic losses due to high piglet mortality. We discovered that gene 7 is an influential virulence factor that evades host immunity by regulating the epitranscriptome, providing a plausible molecular basis for developing novel attenuated vaccine strains. Specifically, as shown in this paper, targeted knockout of gene 7 offers a promising and genetically defined strategy for designing live attenuated vaccines. Furthermore, in addition to its veterinary importance, TGEV is also an important model for understanding coronavirus biology. The mechanism we describe – the mechanism by which viral accessory proteins fine-tune host m6A modifications – may represent a conserved strategy in coronaviruses. Therefore, our work not only contributes to the control of TGEV but also helps improve preparedness for emerging coronaviruses by highlighting RNA modification mechanisms as potential targets for broad-spectrum antiviral interventions.

In summary, our study leverages the power of nanopore direct RNA sequencing to delineate a novel mechanism by which the TGEV accessory protein, gene 7, orchestrates the viral and host epitranscriptomic landscape to promote viral replication. We demonstrate that gene 7 functions as a molecular rheostat, suppressing the m6A writer RBM15 and enhancing the eraser FTO to globally reduce m6A methylation on both host and viral RNAs. This epitranscriptomic dampening is coupled with the suppression of antiviral innate immunity and is essential for optimal viral fitness, as its deletion leads to hyper-methylation, a potentiated host immune response and a severe replication defect. Furthermore, we uncover a gene 7-dependent regulation of viral polyA tail length, adding another layer to its multifaceted role in post-transcriptional control. These findings position viral accessory proteins as key modulators of the RNA modification machinery and reveal the targeting of the m6A pathway as a conserved strategy across coronaviruses. Our work not only advances the understanding of *Alphacoronavirus* pathogenesis but also provides a compelling rationale for the development of broad-spectrum antiviral strategies and live-attenuated vaccines targeting epitranscriptomic regulatory nodes.

## Supplementary material

10.1099/mgen.0.001684 Supplementary Material 1.

10.1099/mgen.0.001684 Uncited Supplementary Material 2.

## Abbreviations

BH-FDR: Benjamini–Hochberg procedure to control the false discovery rate
CI: confidence interval
CPM: counts per million
DEG: differentially expressed gene
DEI: differentially expressed isoform
FDR: false discovery rate
GO: Gene Ontology
gRNA: genomic RNA
m6A: N6-methyladenosine
MF: molecular function
ONT: Oxford Nanopore Technologies
PABP: polyA-binding protein
PR AUC: area under the precision–recall curve
ROC AUC: area under the receiver operating characteristic curve
sgRNAs: subgenomic RNAs
ST: Swine testis
TGEV: transmissible gastroenteritis virus
