# Supplementary material for: Nanopore direct RNA sequencing reveals transmissible gastroenteritis virus epitranscriptomic and transcriptomic landscapes modulated by gene 7
Source: Microb Genom. 2026 Apr 29;12(4):001684. doi: 10.1099/mgen.0.001684 (PMC13135465; doi:10.1099/mgen.0.001684)
Supplement: Supplementary Material 1. [file mgen-12-01684-s001.pdf]

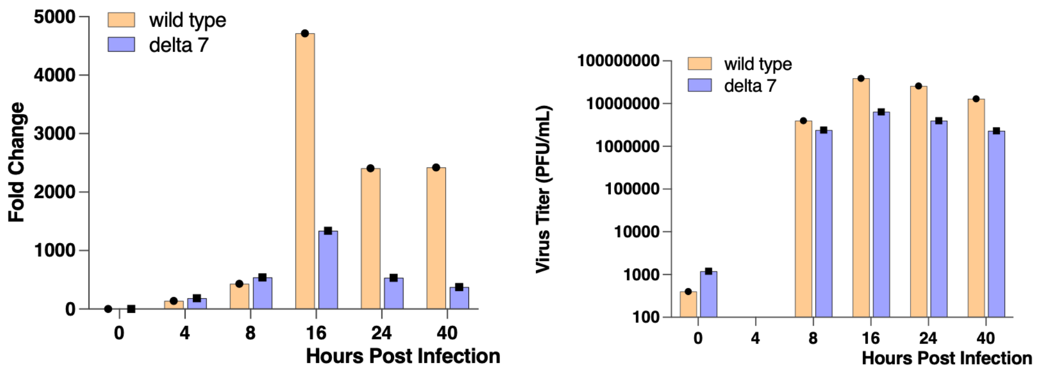

Figure S1. Viral growth kinetics and RNA abundance during a time-course infection. (A) Relative viral RNA abundance (fold change) measured at the indicated hours post infection (hpi) in cells infected with wild type virus or the delta 7 mutant. Values are shown as fold change relative to the 0 hpi time point. (B) Viral titers (PFU/mL) of wild type virus and the delta 7 mutant at the indicated time points post infection. Titers were determined by plaque assay. The data indicate that viral replication reaches a plateau around 16 hpi, which was therefore selected for downstream RNA-based analyses.

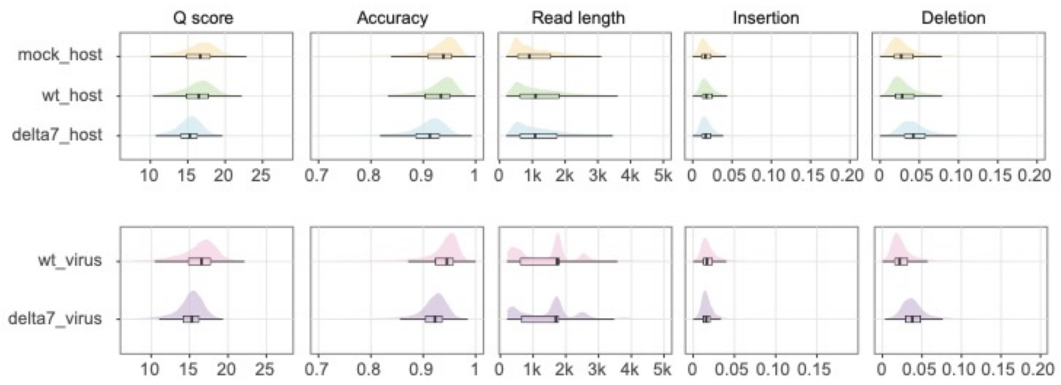

Figure S2. Reads features of host and virus, reads basecalled using Dorado base caller. In the upper row of figures, yellow represents mock data, green represents host data of wt, and blue represents host data of delta7. In the lower row of figures, pink represents virus data of wt while the purple represents virus data of delta7. Read-level Q score, accuracy, read length, insertions, and deletions are calculated based on the mapping results

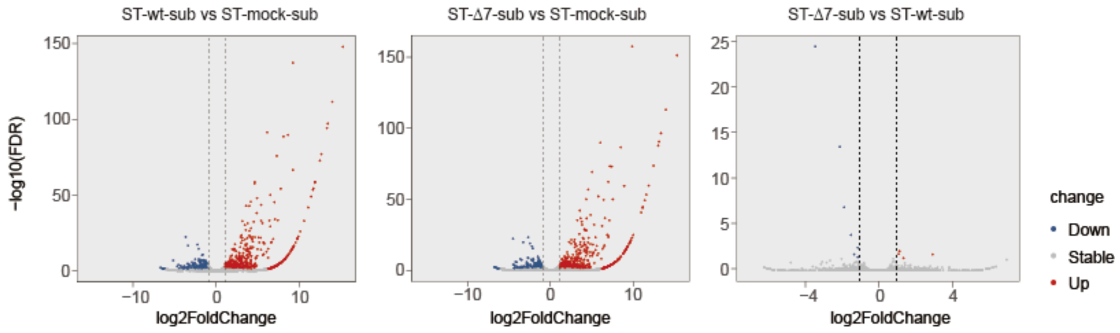

Figure S3. The volcano plots of the subsampled DEGs show a basic trend consistent with Figures 4B, C, and D.

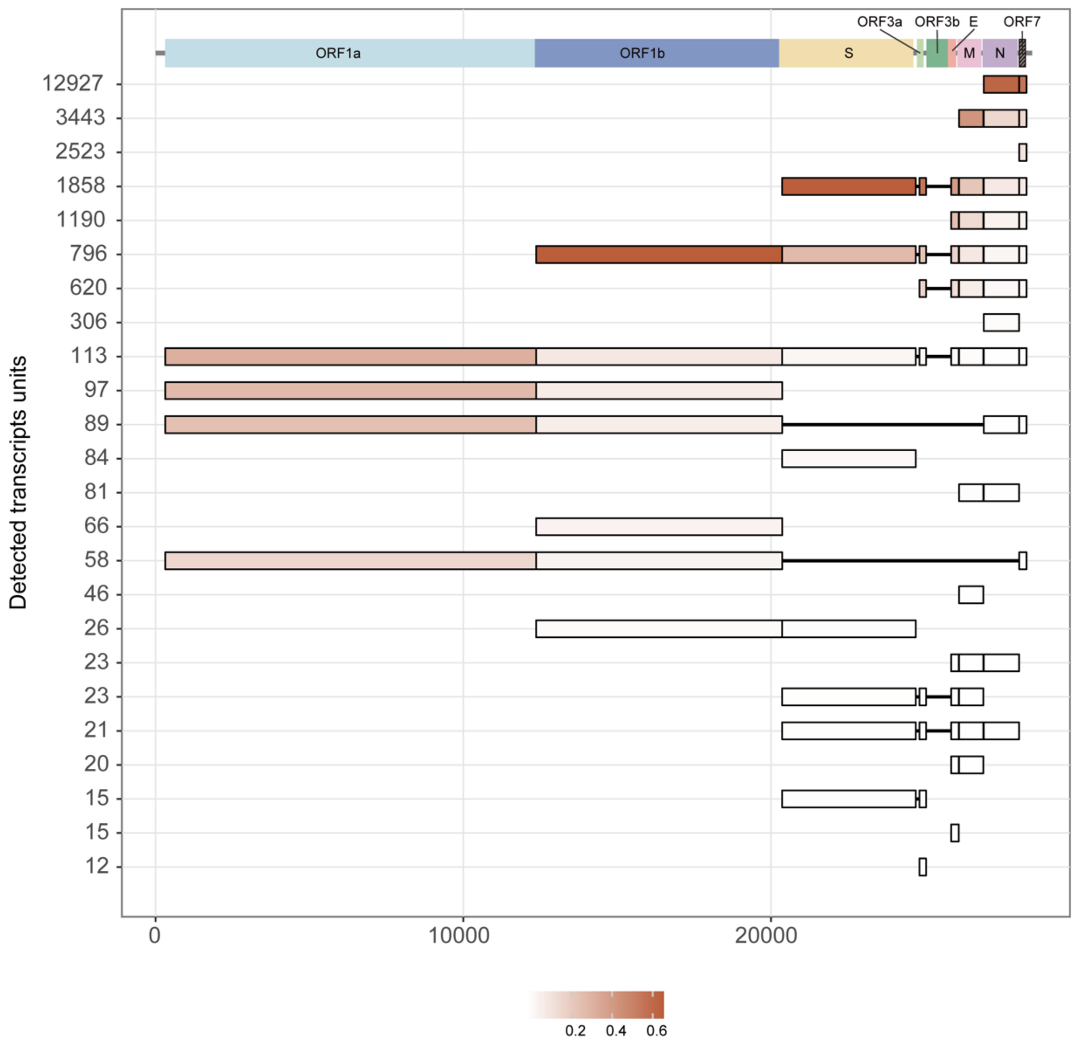

Figure S4. Virus reads mapping result according to genomic and subgenomic RNAs.

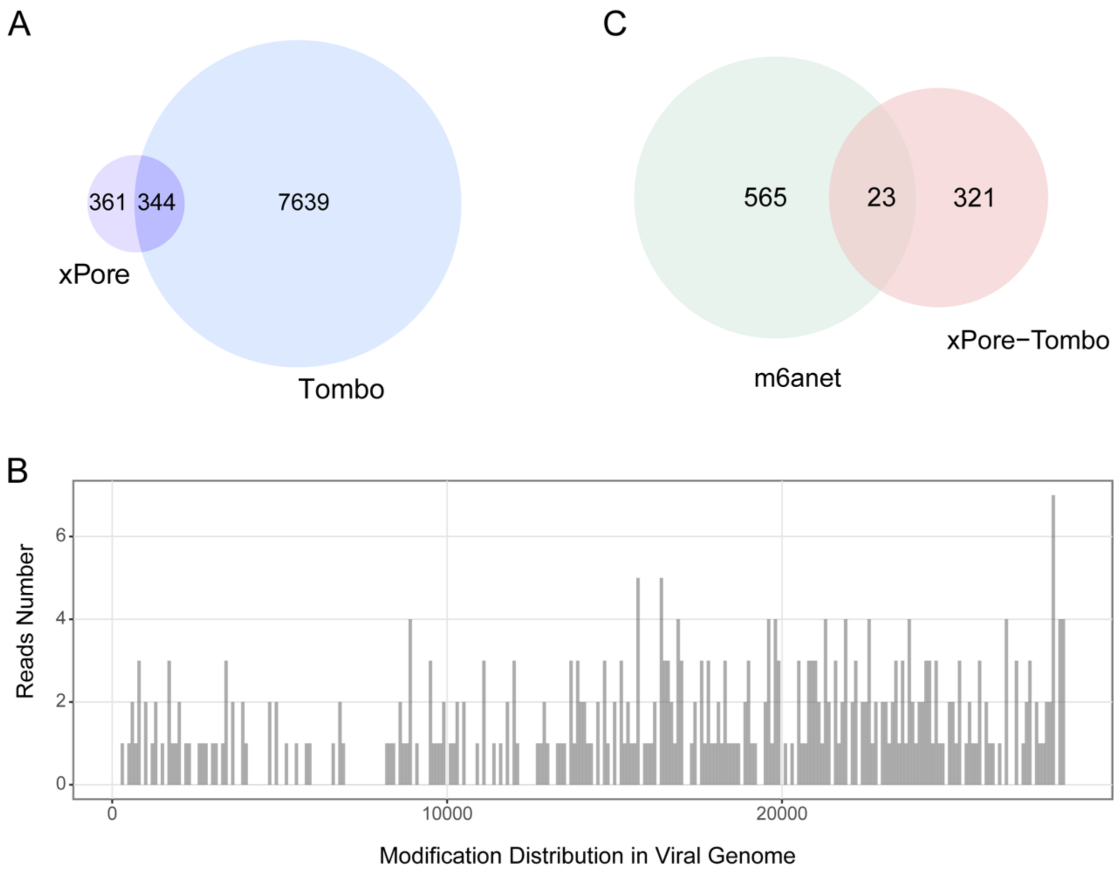

Figure S5. Modified sites detected by xPore and Tombo. (A) Intersection of xPore and Tombo. (B) Distribution of the intersection result of xPore and Tombo. (C) Shared site of m6Anet and intersection result of xPore and Tombo.

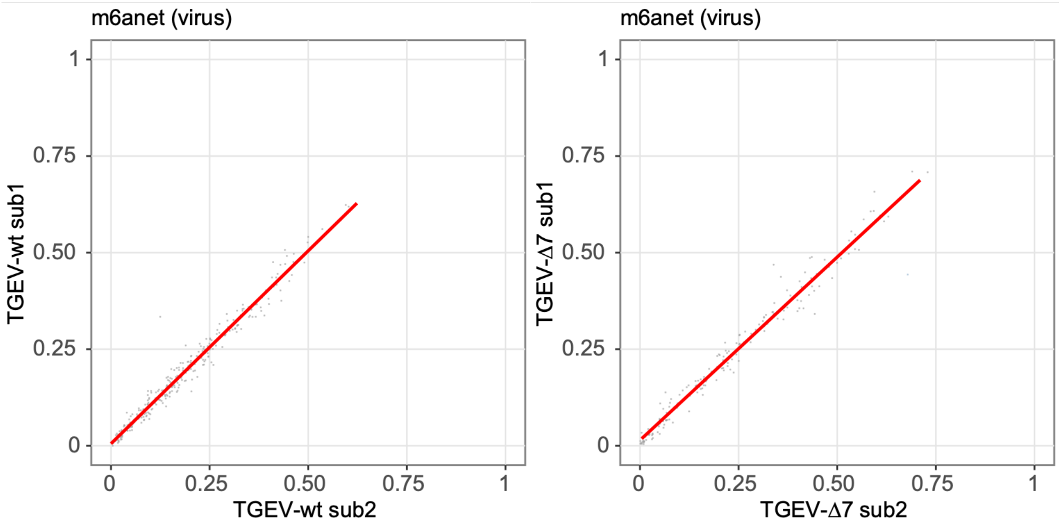

Figure S6. Pearson correlation of the virus's m6Anet output after subsampling, the correlation coefficient of TGEV-wt subsamples is 0.985, and the value of TGEV-Δ7 is 0.988.

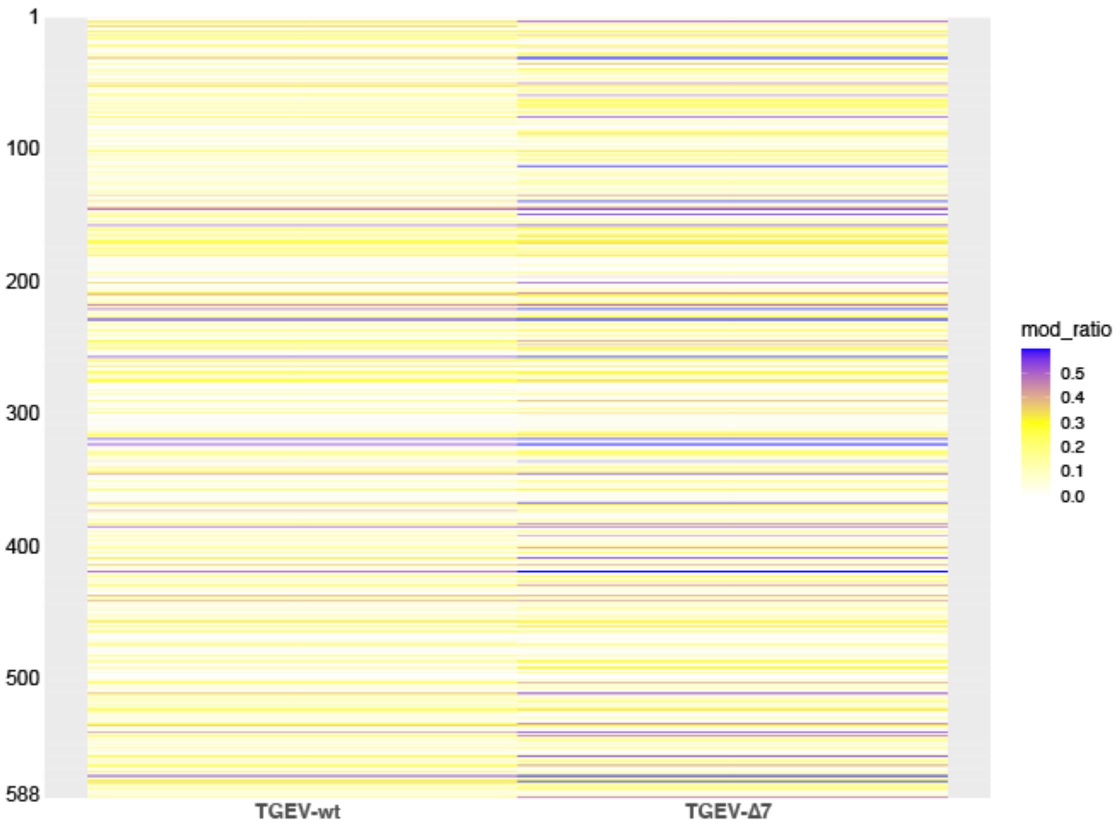

Figure S7. Heatmap comparing m6A modification ratios between two groups. Each row represents a transcript site (numbered sequentially on viral chromosome, not evenly distributed at regular intervals. Specific locations are detailed in Supplementary Material Table S.), and the two columns correspond to TGEV-wt and TGEV-Δ7. Tile color indicates the modification ratio (white = low, yellow = intermediate, blue = high); values are displayed on a 0–0.6 scale (values outside this range are squished to the nearest limit).

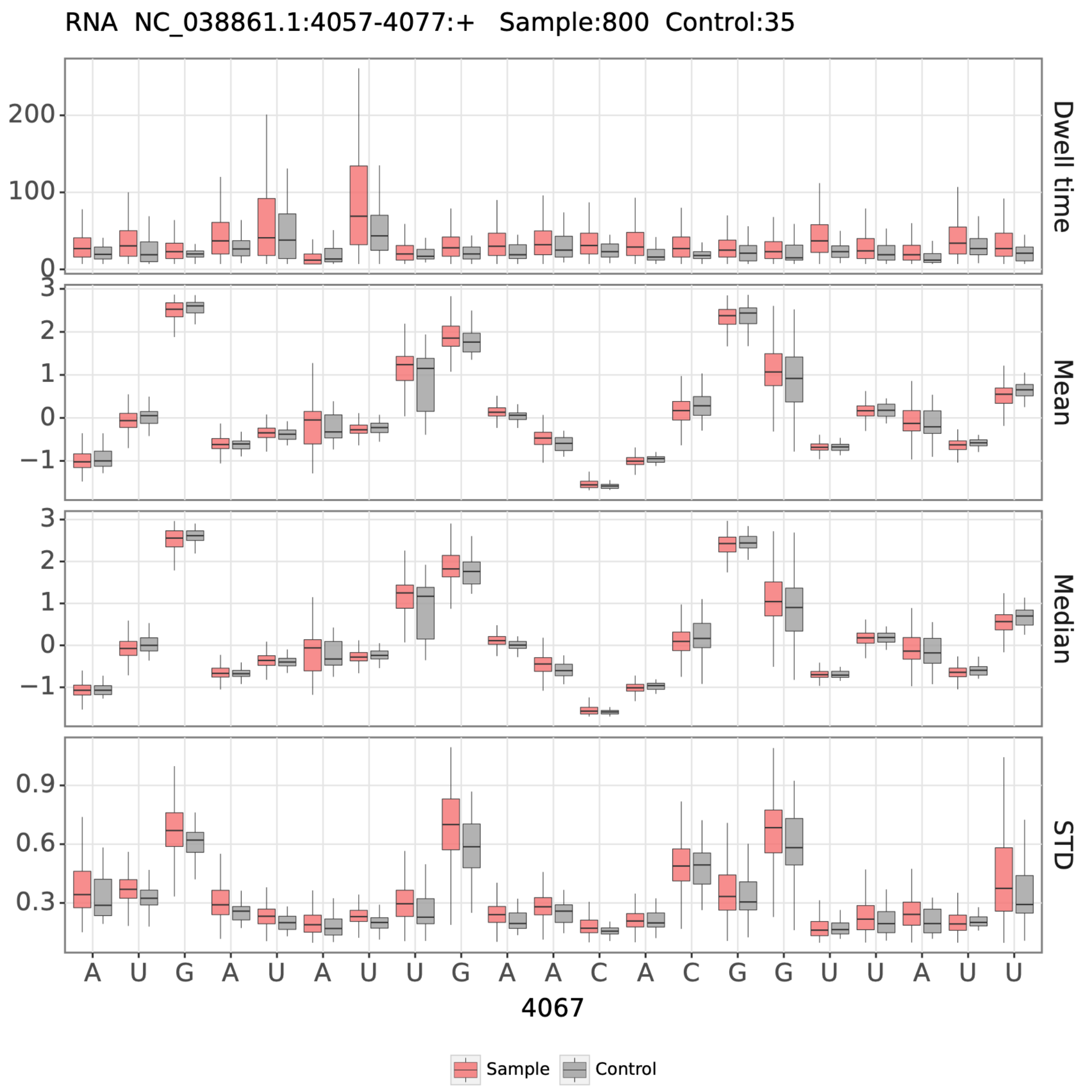

Figure S8. The ONT raw current changes of the m6A site at position 4067.

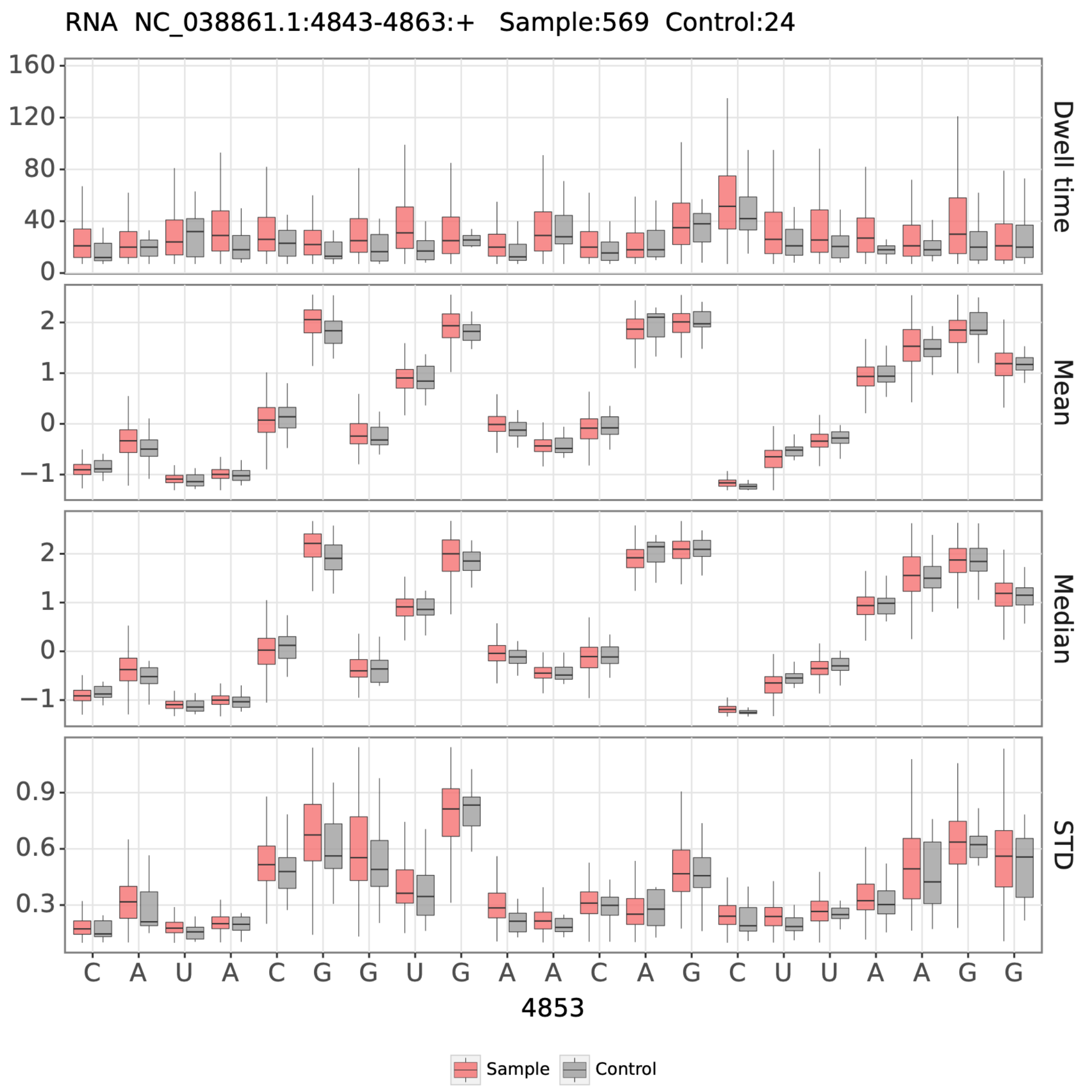

Figure S9. The ONT raw current changes of the m6A site at position 4853.

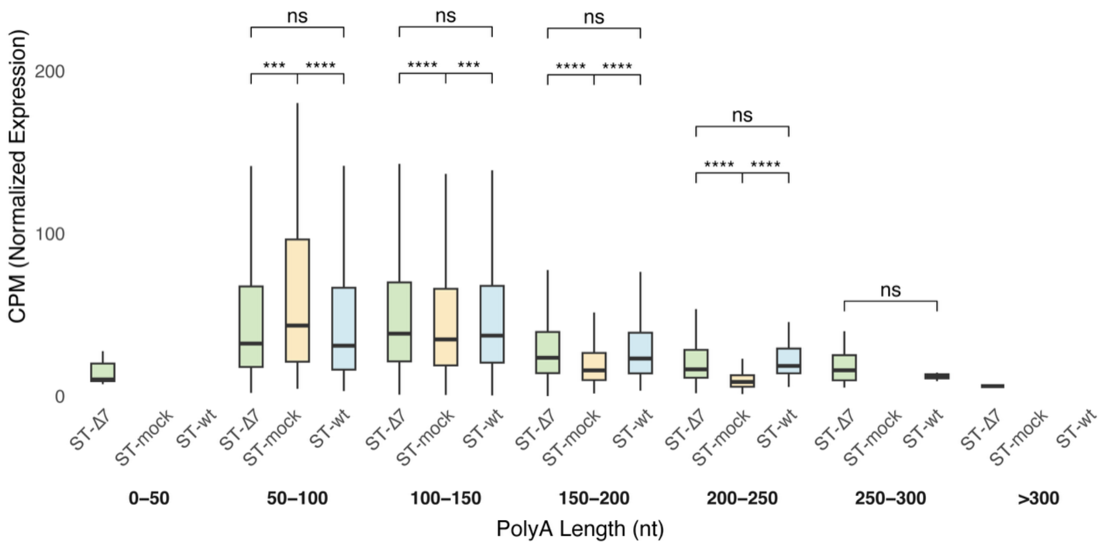

Figure S10. Distribution of host polyA after CPM (50 nt interval). The difference between infected hosts and healthy cells was significant. The polyA tail length of 50-100 nt was the most abundant in mock (healthy ST cells), and both ST-wt and ST-Δ7 showed more distribution in 100-150 nt. However, the loss of TGEV gene 7 did not cause a difference between ST-wt and ST-Δ7.

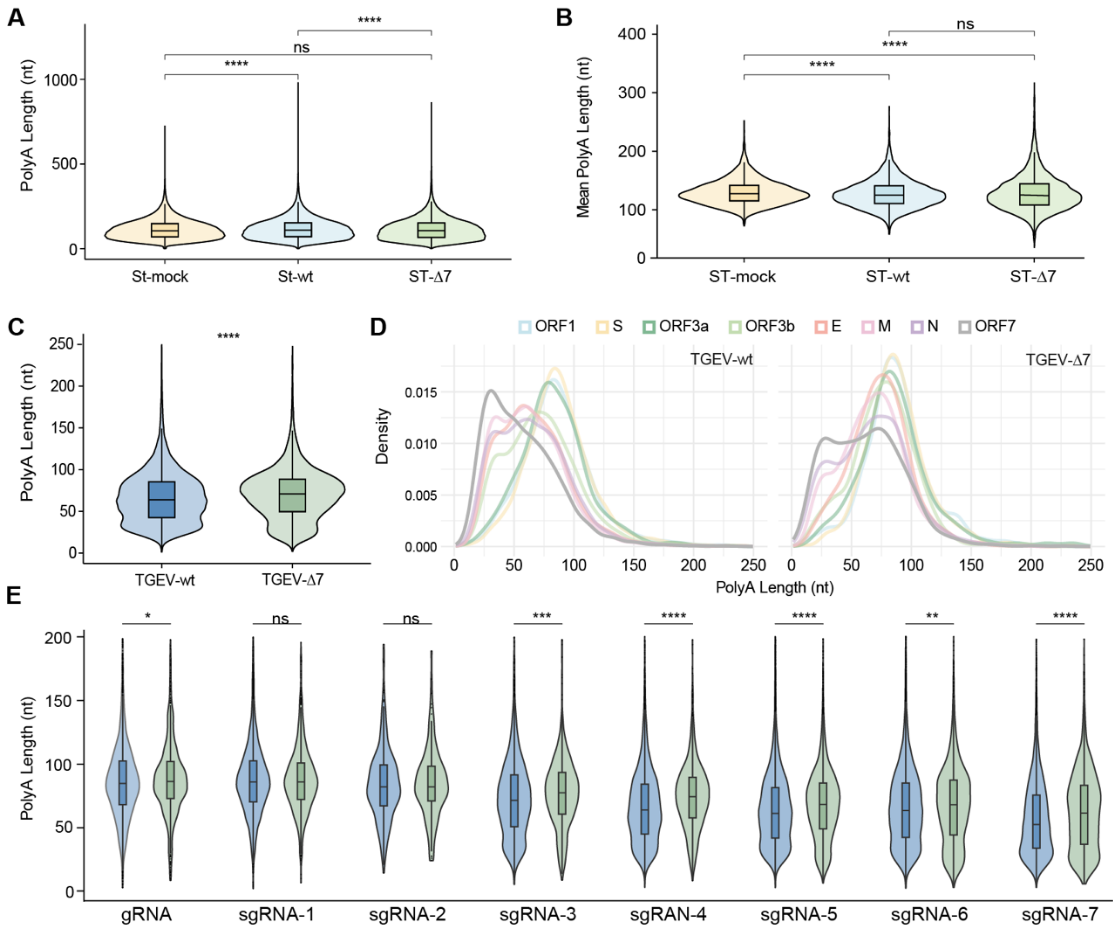

Figure S11. Changes in RNA polyA lengths of viruses. (A) PolyA length distribution of total mRNA from ST-mock, ST-wt, and ST-Δ7. (B) Mean polyA length distribution of filtered genes. (C) PolyA length distribution of all viral RNAs. (D) PolyA length distribution of viral reads after grouping according to genomic and subgenomic transcriptional features. (E) Pairwise comparison of TGEV-wt and TGEV-Δ7 genomic RNA and sgRNAs lengths. Statistical significance was determined by the Mann-Whitney U test (Wilcoxon rank-sum test). Significance levels: ns, not significant ($p \geq 0.05$); *$p < 0.05$; **$p < 0.01$; ***$p < 0.001$; ****$p < 0.0001$.
